# A Database of Plant Heat Tolerances and Methodological Matters

**DOI:** 10.1002/ece3.73813

**Published:** 2026-06-17

**Authors:** Timothy M. Perez, Alyssa T. Kullberg, Evan M. Rehm, Kenneth J. Feeley

**Affiliations:** ^1^ Department of Biology The University of Miami Coral Gables Florida USA; ^2^ Plant Ecology Research Laboratory PERL, School of Architecture, Civil and Environmental Engineering EPFL Lausanne Switzerland; ^3^ Community Ecology Unit Swiss Federal Institute for Forest, Snow and Landscape Research WSL Lausanne Switzerland; ^4^ USDA Forest Service Northern Research Station Morgantown West Virginia USA

## Abstract

Plant heat tolerance data are increasingly valued for their potential to help increase our understanding of species' responses to extreme temperatures, but these efforts are hindered by methodological inconsistencies and missing contextual information. To address this issue, we collated data on heat tolerance estimates and key sources of variation attributable to taxonomy, methods, geography, and cultivation. The resultant resource is designed to catalyze more rigorous and ecologically meaningful syntheses by enabling researchers to identify, account for, and test the drivers of variation in plant heat tolerances and their consequences. We collected heat tolerance data for terrestrial plants using traditional literature searches and from previously existing databases while expanding the scope of data collected to account for taxonomic, methodological, biogeographic, cultivation, and bibliometric biases. We collected > 3100 heat tolerance records reported in degrees Celsius, from primarily vascular plants encompassing > 1700 taxa, > 1000 genera, and > 200 families from years 1935–2024. Our database more than doubles the species and more than triples the number of records for heat tolerances compared to other databases. Our database is global in scope, but we highlight the lack of standardized methods, undersaturated taxonomic sampling, and underrepresented geographic regions.

## Graphical abstract

We collected > 3100 heat tolerance records reported in degrees Celsius, from terrestrial plants encompassing > 1700 taxa, > 1000 genera, and > 200 families from years 1935–2024. Our database is global in scope, but we highlight a lack of standardized methods, undersaturated taxonomic sampling, and underrepresented geographic regions.

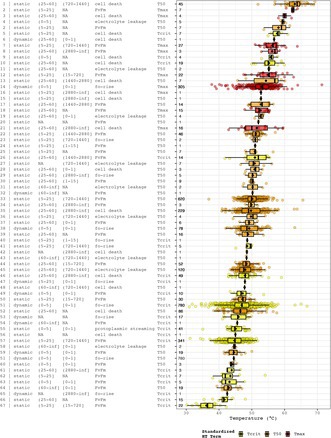

## 1 Introduction

Given the fundamental importance of photosynthesis in plant function, photosynthetic tissues are commonly used to assess plant heat tolerance. Plant heat tolerances are often reported as the temperatures that cause significant reductions in normal biological or physiological functions and have potential applications in breeding heat tolerant crops (Bita and Gerats 2013; Langridge and Reynolds 2021) and natural resources management (Allen et al. 2010; Rehfeldt et al. 2014). In ecological contexts, plant heat tolerances are often touted for their potential value for revealing and predicting plant responses to extreme temperatures, but their relevance to higher‐order processes like growth, demographic rates, species distributions, and carbon sequestration remains uncertain.

The difficulties in drawing mechanistic ecological conclusions or predicting organismal vulnerability to thermal extremes from heat tolerances is partly due to a lack of standardized methods (Geange et al. 2021). Although assays of plant heat tolerances generally include similar procedures, differences within these steps can cause variation in heat tolerance estimates (Perez et al. 2021; Hauck et al. 2025). Variation in methods has long been known to make direct comparisons of heat tolerances difficult among studies (Lange 1965).

Different physiological responses used to measure heat tolerances with otherwise identical procedures can also result in contrasting heat tolerance estimates and subsequent inferences. For example, cell vitality, cell membrane stability, and photosystem II (PSII) function are all physiological traits commonly assayed in leaves and other photosynthetic tissues to estimate heat tolerance. Cell death indicates leaf tissue necrosis and is generally quantified by visual inspection including staining, microscopy techniques, and estimating the ratio of dead to living tissue (e.g., Onwueme 1979; Chen et al. 1982; Larcher et al. 1989). High temperatures that cause cell and organelle membrane damage can be measured with electrolyte leakage. The leakage of cellular contents into a solution is typically measured using changes in electrical conductivity. Chlorophyll *a* fluorescence may also signal the disruption of thylakoid membranes in chloroplasts caused by high temperatures (Schreiber et al. 1976; Wahid et al. 2007; Zhu et al. 2024). However, chlorophyll *a* fluorescence is most commonly used to assess processes closely related to photosystem II (PSII) function, which is temperature sensitive (Krause and Santarius 1975; Baker 2008).

Fluorescence of the PSII complex that is induced using a minimum quantity of light while maintaining no electron transport is termed F0 or fo (and fo hereafter), and can increase as photosynthetic tissues are damaged. Increasing fo signals the closure of PSII reaction centers. A large pulse of light can completely reduce all PSII's reaction centers and cause a peak in fluorescence termed the maximum fluorescence (Fm). The difference between Fm and fo is termed variable fluorescence (Fv). The parameter Fv/Fm (FvFm) provides an estimate of the maximum quantum efficiency of PSII. Impairment of PSII function is commonly measured using relative changes in fo or FvFm parameters.

Cell death, electrolyte leakage, and chlorophyll fluorescence reveal physiological information at increasingly finer levels of cellular function while decreasing in biological complexity. For example, the loss of PSII function doesn't necessarily indicate loss of cell vitality or membrane integrity. Conversely, the function of cell membranes and PSII have little physiological relevance at cell death. Consequently, these heat tolerances represent different physiological processes and should not be considered interchangeable.

Methodological inconsistencies and physiological nuances among studies may be inconspicuous, but are important considerations for avoiding misuse of, or misinterpretations from, heat tolerance data. For example, despite limited evidence, the heat tolerance of photosystem II has been conflated with the temperature causing leaf and tree canopy death, and has been used to make predictions about global forest mortality and ecosystem tipping points (Doughty et al. 2023; Winter et al. 2024). Similarly, some studies have compiled heat tolerance estimates from differing physiological processes—for example, electrolyte leakage and photosystem II function (Araújo et al. 2013) or respiration and carbon assimilation (Lancaster and Humphreys 2020)—to infer broad patterns in plant evolution and vulnerability to climate change. However, combining these different physiological mechanisms confounds clear inferences regarding broader ecological and evolutionary processes.

Clarifying the methods used to estimate heat tolerance is essential for drawing meaningful conclusions about underlying physiological mechanisms and for linking them to broader phenomena such as organismal performance and fitness. We compiled a database of plant heat tolerance estimates for photosynthetic tissues and included other variables to help disambiguate several major sources of variation among studies. Our database includes types of (1) taxonomic, (2) methodological, (3) biogeographical, and (4) cultivation data as a resource for researchers. We describe the data we collated in our database, summarize it, and briefly highlight the effects that methodological variation can have on heat tolerance estimates. Our goal is to catalyze heat tolerance research that links physiological to higher‐order ecological and evolutionary processes.

## 2 Methods

### 2.1 Data Acquisition

To assemble our database, we identified peer‐reviewed articles following forward and backward citation procedures and cross‐referencing other datasets that report heat tolerance for studies published up until year 2024. In backward citation procedures, references from existing publications are used to identify new references that are then evaluated for inclusion. In forward citation procedures, references to an existing publication are used to identify new references that are then evaluated for inclusion. We used existing papers in our libraries to initiate our search and Google Scholar as our method for forward citation procedures. We included data from the articles found with our citation searches if they contained heat tolerances that were (1) reported for terrestrial plants, (2) in units of temperature, (3) for photosynthetic tissues, and (4) did not quantify carbon assimilation or respiration, which are already treated in databases elsewhere (e.g., Kumarathunge et al. 2019; Atkin et al. 2015). A flowchart illustrating the main steps we used to determine the eligibility of data to include in our database is provided in our Section 1: Appendix S1.

The majority of articles and datasets that we included were located by T. M. Perez (2019). This dataset was also established through forward and backward citation searches over the course of dissertation research, and contributed 69 references corresponding to 941 plant species and 1396 heat tolerance records (Table 1). This dataset was then cross‐referenced against the GlobTherm (Bennett et al. 2018) and Lancaster and Humphreys (2020) (hereafter GlobTherm and Lancaster and Humphreys, respectively) to locate articles not currently in the T. M. Perez (2019) dataset.

**TABLE 1:** Dataset summaries.

Dataset	Dataset summary		
	References (*n*)	Unique species (*n*)	Records (*n*)
T. M. Perez (2019) (baseline)	69	941	1435
Present study (baseline)	131	1768	3197
Lancaster and Humphreys (2020) (Tmax)	37	689	964
GlobTherm (Streptophyta + Tmax)	12	73	73

*Note:* Lancaster and Humphreys (2020) restricted to Tmax rows. GlobTherm restricted to Streptophyta with Tmax.

Articles not already within the T. M. Perez (2019) dataset were screened for compatibility. To ensure the consistency and fidelity of data in our database, we collected metadata directly from compatible articles instead of from Globtherm's and Lancaster and Humphrey's datasets. This was necessary since our initial screenings of these datasets identified omissions of heat tolerances for some species, and details on methods, or at times included what we considered to be incompatible methods from the original source material. A diagram illustrating how references from other databases were incorporated into our database and overlap with one another is provided in Figures S1 and S2. A list of comparisons across databases are found in Tables S1 and S2. Our description of the heat tolerance metadata that we collected and our procedure for collating it is described below.

### 2.2 Taxonomic Data and Description

Taxonomic data were recorded for each heat tolerance estimate in our database (see Section 2.2.1). We updated taxonomic data (see Section 2.2.2) from the original sources to account for any changes in nomenclature.

#### 2.2.1 original_species

The original_species column contains the original genus and species names from the source of the heat tolerance estimate. Some of the reported taxonomic classifications in the literature are outdated or contain orthographic errors.

#### 2.2.2 updated_species

To standardize the taxonomic information in the database, we used the “wcvp_match_names” function in the “rWCVP” R package (Brown et al. 2023) to update the *original_species* data according to the World Checklist of Vascular Plants (Govaerts et al. 2021). Then we used the “resolve_multi” function to select the best fitting name when multiple matches were present for a single supplied taxon. Lastly, we inspected and corrected all rows where the final match was not exact, and manual corrections were made. Genus and family names were entered when higher‐resolution taxonomic data were not evident.

#### 2.2.3 wcvp_authors

The wcvp_authors column contains the taxonomic authorities for a given species as recorded in the updated_species data according to the WCVP database.

#### 2.2.4 family

The family column contains the plant family associated with the updated_species names.

### 2.3 Methodological Data and Description

We collected methodological data associated with each estimate of heat tolerance (Section 2.3.1). These data included the type of heating treatment (Section 2.3.2), the duration of heat treatment (Section 2.3.2.1), the length of recovery time following heat treatment (Section 2.3.2.3), the physiological method used to measure the response to heat treatment (Section 2.3.3) and the metric used to estimate heat tolerance (Section 2.3.3.1). To simplify the variation across studies, we categorized the original duration of heat treatment (Section 2.3.2.2), the recovery time following heat treatment (Section 2.3.2.4), and the original term used for each heat tolerance (Section 2.3.3.2). For the standardized categories, we use standard interval notation, where square brackets indicate that the endpoint is included and parentheses indicate that the endpoint is excluded. For example, “[” indicates greater than or equal to the lower bound, and “)” indicates strictly less than the lower bound.

#### 2.3.1 HT

We extracted the maximum heat tolerance metric that was reported per species per location within each article and recorded it in the *HT* column. Heat tolerance estimates were obtained from the text, supplemental data, or digitized from figures and were converted to °C when necessary. Digitized data were obtained using the plot digitizer web app (https://plotdigitizer.com/about).

#### 2.3.2 static_dynamic

The type of heating method used to study plant heat tolerances. Values are either “static” or “dynamic”. Static refers to heat treatments that expose tissues to a given temperature for a fixed period of time. Dynamic refers to heat treatments that expose tissues to temperatures that change at a constant rate, often raising temperature to a high‐temperature target over a given period of time (Lutterschmidt and Hutchison 1997). Both static and dynamic heat methods may use a variety of heat sources (e.g., peltier devices, water baths, heating plates), but these are not provided in our database.

##### 2.3.2.1 original_duration

The duration of heat treatment used to estimate heat tolerances in minutes for static heating assays. Some research suggests that increasing the duration of heat treatment causes decreases in the estimates of heat tolerances (Colombo and Timmer 1992; Cook et al. 2024). However, it is possible this pattern varies with the accompanying methodological procedures. Similarly, Arnold et al. (2021) report that the heating rate of dynamic heating methods can bias heat tolerance estimates. We recorded these dynamic heating rates in degrees per second (°C s^−1^).

##### 2.3.2.2 duration_category

The heat treatment duration was categorized as follows: > 0 and ≤ 5 min is abbreviated as (0–5]; > 5 and ≤ 25 min is abbreviated as (5–25]; > 25 and ≤ 60 min is abbreviated as (25–60]; > 60 min or more is abbreviated as (60‐inf]; and “NA” is recorded if no duration was specified. These intervals were selected to distribute heat tolerances into groups that would facilitate comparisons that are also easily implemented by practitioners.

##### 2.3.2.3 original_recovery

The recovery time recorded as the minutes elapsed following removal of tissues from heat treatment until the damage was quantified. Recovery time can influence heat tolerances because some physiological functions can recover following heat treatments (Havaux 1993; Kitao et al. 2000; Krause et al. 2010). Recovery times were recorded as zero for heat tolerance estimates determined with dynamic heating methods since in these cases the damage is assessed simultaneously to treatment.

##### 2.3.2.4 recovery_category

The heat treatment recovery times from the original_recovery data recorded as follows: 0 and ≤ 1 min is abbreviated as [0–1]; > 1 and ≤ 15 min is abbreviated as (1–15]; > 15 and ≤ 720 min is abbreviated as (15–720]; > 720 and ≤ 1440 min is abbreviated as (720–1440]; > 1440 and ≤ 2880 min is abbreviated as (1440–2880]; > 2880 min or more is abbreviated as (2880‐inf]; and “NA” is recorded if no recovery time was specified. These intervals were selected to distribute heat tolerances into groups that would facilitate comparisons that are also easily implemented by practitioners.

#### 2.3.3 Method

The different physiological response variables measured to assess damage following heat treatments. We recorded the physiological response variable associated with each heat tolerance estimate in the method column of our database. We collected heat tolerance data and recorded any associated methods as long as they met the criteria described above.

##### 2.3.3.1 original_term

Once tissue damage is quantified it can be used to estimate the temperature that causes a predefined level of physiological impairment. There are several different metrics of heat tolerances that have been reported, but these tend to correspond to the temperatures that cause the first signs of impairment, cause a 50% change in the physiological response variable, or cause a near complete change in the physiological response variable. We recorded the original term for the heat tolerance from the source article in the metric columns of our database.

##### 2.3.3.2 HT_standardized

The original terms reported from studies were categorized as Tcrit if the reported metric calculated represents the lowest temperature reported that leads to an initial measurable or significant reduction in the physiological response measured following heat treatment, T50 if the metric calculated represented a 50% change in the physiological response measured following heat treatment, or Tmax if the metric calculated represented a maximal reduction in the function of the physiological response variable being measured. The standardized metrics are recorded in the HT_standardized column of our database. Importantly, these categories are meant to refer to heat‐induced *change* in a given biophysical response variable relative to control or ambient conditions and are independent from other methodological considerations.

### 2.4 Biogeographical Data

Geography may influence heat tolerances through effects of climate, microclimate (Feeley et al. 2020; Perez and Feeley 2020), acclimation, or local adaptation (Knight and Ackerly 2003; Zhu et al. 2018). We recorded provenance and growth location information when possible in an attempt to partially account for these effects. Provenance data indicates the location that the plant tissues originated (see Sections 2.4.1 and 2.4.2), and growth location indicates where plants were grown before tissue were sampled for measurement (see Sections 2.4.3 and 2.4.4). We also recorded if plant tissues were grown in outdoor environments or controlled environments in our cultivation data (Section 1, 2.5).

We recorded the coordinates for provenance or growth sites when they were reported. These coordinates were reviewed and altered for more accuracy when necessary. For example, coordinates that were rounded at the time of reporting leading to inaccuracies (i.e., occurring in water) were altered when additional information was present that would allow more precise geolocation. We used Google Maps (https://www.google.com/maps) and GEOLocate webapps (https://www.geo‐locate.org/web/WebGeoref.aspx) to determine approximate coordinates of definitive locations or landmarks when they were provided but geographic coordinates were not. When only vague general locations were provided, we inferred probable approximate coordinates using the information provided from source articles and accessible information like vegetation cover, elevation, and our expert judgment. If we could not confidently determine the geolocation within 10 km of its most likely ecosystem collection location, no coordinate was recorded.

We mapped the growth site locations for all records that also reported sample provenance data to the Intergovernmental Panel on Climate Change (IPCC) Working Group I Reference Regions (Allen et al. 2018) and Whittaker biome space to characterize the biogeographical distribution of the data represented within our database. The IPCC regions represent sub‐continental areas used for broad‐scale biophysical climate modeling in the Coupled Model Intercomparison Project (Iturbide et al. 2020), and Whittaker biomes characterize biomes based on coarse temperature and precipitation gradients. Data were mapped to the Whittaker biomes based on the *plotbiomes* R package (Ștefan and Levin 2018). Records with no geographic data were not mapped to IPCC regions or Whittaker biomes.

#### 2.4.1 provenance_latitude

The provenance_latitude column records the latitude in decimal degrees where plant material originated prior to heat tolerance measurement.

#### 2.4.2 provenance_longitude

The provenance_longitude column records the longitude in decimal degrees where plant material originated prior to heat tolerance measurement.

#### 2.4.3 growth_site_lat

The growth_site_lat column records the latitude in decimal degrees where plants were grown prior to heat tolerance measurement.

#### 2.4.4 growth_site_lon

The growth_site_lon column records the longitude in decimal degrees where plants were grown prior to heat tolerance measurement.

#### 2.4.5 location_name

The location_name column records the name of the location where heat tolerance measurements were made and was used to gather provenance or growth site information where applicable.

### 2.5 Cultivation Data

Our database focuses on undomesticated plant species but does contain data for some domesticated and cultivated species. The distinction between wild and domesticated or cultivated species is likely relevant to researchers with differing expertise, and may be useful for understanding the effects of selective breeding on heat tolerance. We recorded the cultivation status of each species (Section 2.5.1) and basic information on the growing conditions of the plants used to estimate heat tolerances where possible (Section 2.5.2).

#### 2.5.1 Agricultural_sp

The cultivated and or agricultural status of species measured. Species were considered agricultural or cultivated if they are widely seen as commercial crops, commodities, crops grown for sustenance, or ornamental purposes. In some cases, the original reference provided information to help determine if the specific individuals used were of cultivated varieties. We cross‐referenced all species in our database against the Crop Wild Relative (CRW) database, which contains scientific names of cultivated plants (Crop Wild Relative Occurrence Data Consortia 2024). Species considered crops are recorded as “Y” indicating yes, or “N” indicating no crop status.

#### 2.5.2 growing_conditions

The growing conditions the plants were grown in prior to heat tolerance assessment. These data include greenhouse, common garden, growth chamber, or in situ designations. Importantly, we do not include information about soil, water, or temperature, or light regimes, which can all influence heat tolerances.

### 2.6 Bibliometric Data

Full citations for each study from which heat tolerance estimates and their associated metadata were obtained (Section 2.6.1).

#### 2.6.1 Reference

The reference column contains the authors, publication date, study title, and journal or analogous information for each heat tolerance estimate.

### 2.7 Correlations Among Methods

We conducted pairwise Pearson correlation analyses between all combinations of heat tolerance measurement methods to understand which physiological responses may exhibit coordinated temperature thresholds. We performed correlation tests using heat tolerances from two different full methods. We considered full methods as the combination of heating type (static_dynamic), heating duration (duration_standardized), recovery period (recovery_standardized), method of physiological assessment (method), and standardized term (HT_standardized) categories associated with each heat tolerance record. Heat tolerances for any species with more than one record per unique full method were averaged before correlations among full methods were tested. Correlations were calculated only when at least five species were shared between methods. We recorded the Pearson correlation coefficient (*r*), associated *p*‐value, and the number of overlapping species (*n*) for each method pair. Correlations were performed using base *R*'s cor.test function and *p*‐values were corrected using the Benjamini & Hochberg (BH) correction methods (R Core Team 2024). These values were compiled into matrices representing correlation strength, significance, and sample size, respectively. We did not account for the potential effects of biogeography, or cultivation.

## 3 Results

### 3.1 Data Acquisition

Our final dataset contained 3197 heat tolerance records; this is three times as many records as the Lancaster and Humphreys dataset, and over 43 times the number of records for terrestrial plants as the GlobTherm dataset (Table 1). Our heat tolerance records correspond to 1768 taxonomic entities, which is over twice as many species as the Lancaster and Humphreys (2020) dataset, and over 23 times the number of records for terrestrial plants in the Globtherm dataset (Table 1). Notably, our database contains 91 references corresponding to 2181 records of heat tolerances for 1231 species that are not present in other datasets.

Our data collection procedure led to considerable differences in the number of species and records in our final database compared to those of GlobTherm and Lancaster and Humphreys. For example, of the 11 references common to both datasets, 72 species and 72 records were reported from GlobTherm while we reported 93 species and 239 records (Table 2). Lancaster and Humphreys' dataset reported 637 species and 856 records while we reported 622 species and 905 records for the same 30 references (Table 2). Reference‐level explanations for differences in the number of species and heat tolerance records between our database, the GlobTherm, and Lancaster and Humphreys datasets are provided in Tables S1 and S2.

**TABLE 2:** Pairwise overlaps between datasets (counts within each overlap).

Pair	Dataset	Overlap summary		
		References (*n*)	Unique species (*n*)	Records (*n*)
Lancaster and Humphreys (2020) ↔ T. M. Perez (2019)	Lancaster and Humphreys (2020) (overlap with T. M. Perez 2019)	16	566	716
	T. M. Perez (2019) (overlap with LH)	16	545	778
GlobTherm (Streptophyta + Tmax) ↔ T. M. Perez (2019)	GlobTherm (Streptophyta + Tmax overlap with T. M. Perez 2019)	7	63	63
	T. M. Perez (2019) (overlap with GlobTherm)	7	81	222
Lancaster and Humphreys (2020) ↔ Present study	Lancaster and Humphreys (2020) (overlap with Present study)	30	637	856
	Present study (overlap with LH)	30	622	906
GlobTherm (Streptophyta + Tmax) ↔ Present study	GlobTherm (Streptophyta + Tmax overlap with Present study)	11	72	72
	Present study (overlap with GlobTherm)	11	93	239

*Note:* Counts reflect the subset defined by shared references in each pair. Lancaster and Humphreys (2020) restricted to Tmax rows. GlobTherm restricted to Streptophyta with Tmax.

### 3.2 Taxonomic Data

**FIGURE 1:**
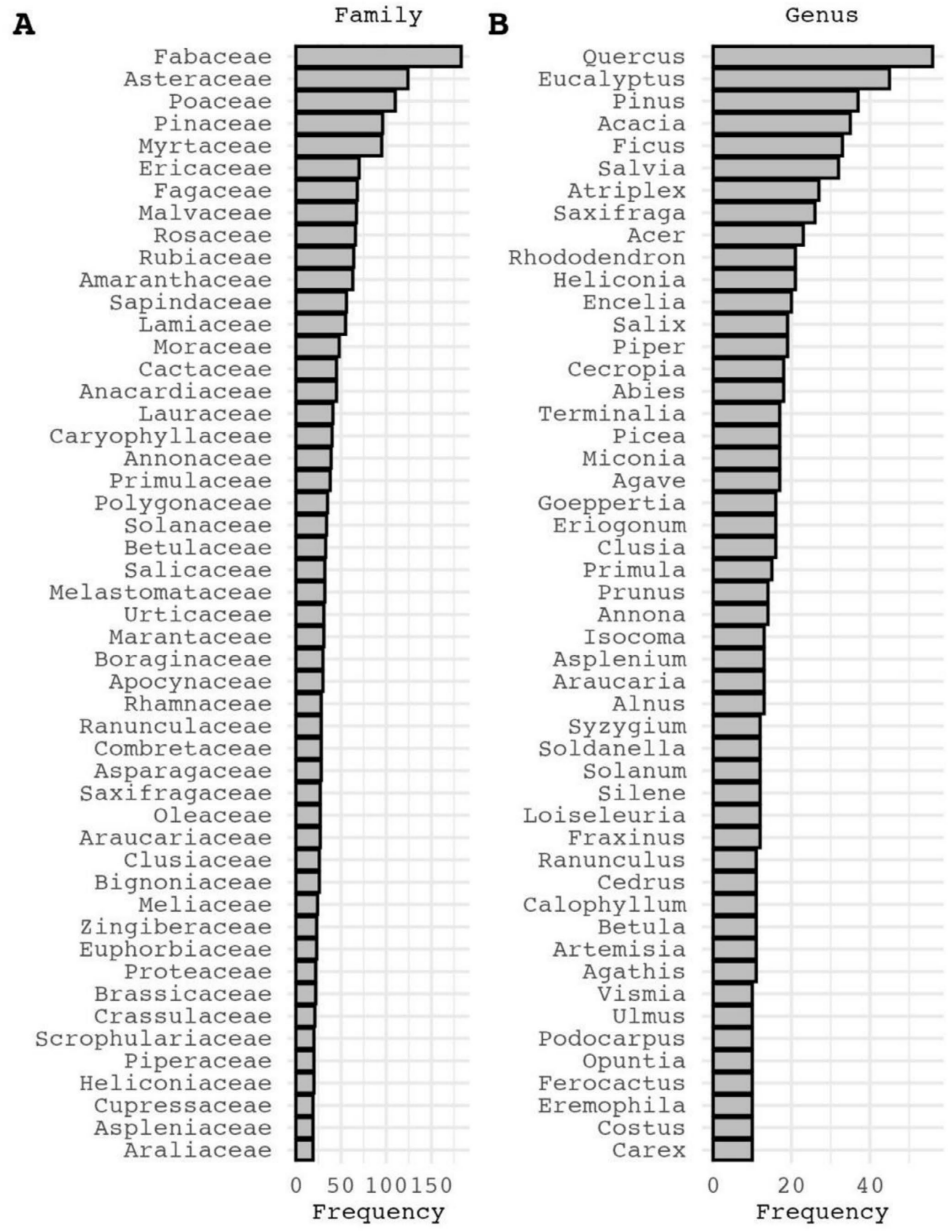
(A) The 50 most commonly reported plant families in the database. (B) The 50 most commonly reported plant genera in the database.

Our database represents heat tolerance estimates belonging to 1768 different taxa classified at the species, genus and family levels. These data correspond to 1001 genera and 250 plant families. The heat tolerances for the 50 most commonly reported families and genera are depicted in Figure 1A,B. The species in our database represent predominantly vascular plants, but include some moss species. A stable version of the database present in this manuscript is stored in https://doi.org/10.5061/dryad.cjsxksnj2. Updated versions of this database will be hosted on https://tmoreperez.github.io/heat‐tolerance‐database/.

### 3.3 Methodological Data

**FIGURE 2:**
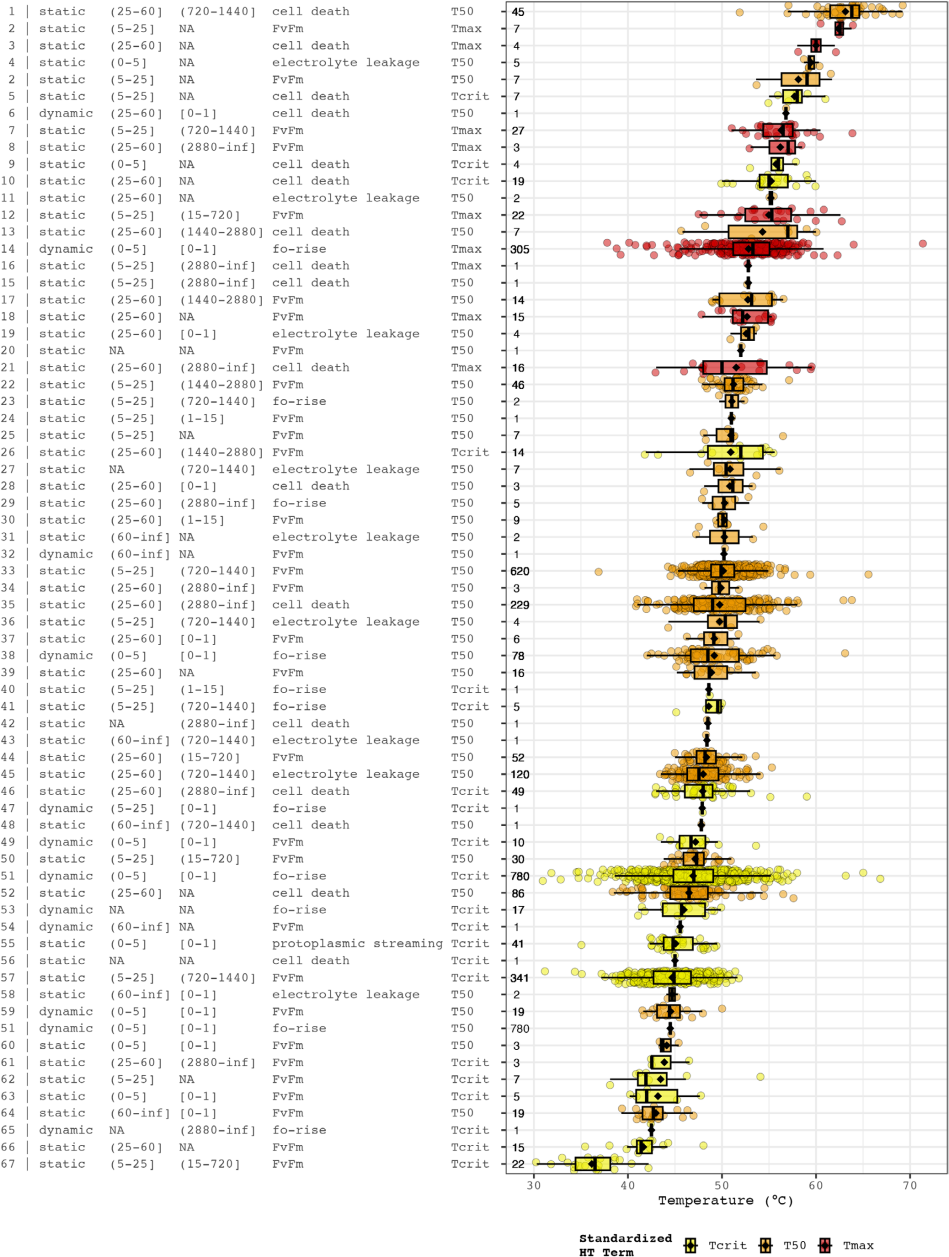
Boxplot of 3197 heat tolerance estimates colored by standardized HT metric (i.e., Tcrit, T50, or Tmax) across 67 distinct methods ordered by mean (black diamonds). Numbers to left and right correspond to distinct methods and number of records, respectively. Numbers on *y*‐axis correspond to distinct method reported in Table S4.

We recorded dynamic versus static heating methods, heat treatment duration, recovery durations, physiological response variables, and the term used to report each heat tolerance. The original reported metadata associated with each heat tolerance represented 139 distinct methods and terms among 131 studies we used to gather data. Our categorizations and standardization procedure resulted in 67 distinct methods. Data from these standardized methods are depicted in Figure 2 with a total number of records associated with each. We noted that 59 different terms were used to describe the heat tolerances that we placed into one of three categorical metrics (Table S3), and that 17 methods represented by a single record of heat tolerance.

#### 3.3.1 HT

Our database contains heat tolerances that are summarized in Figure 2. Descriptive statistics associated with Figure 2 are also included in Table S4, which includes the full complement of methodological metadata for each numbered category. For example, group 1 with the highest mean heat tolerance of 63.1°C represents species that were statically heated for durations of 25–60 min, allowed to recover for 720–1440 min, before having cell death quantified and used to calculate the T50 metric.

#### 3.3.2 static_dynamic

Our database consists of 1983 (62%) heat tolerance records estimated using static heating methods and 1214 (38%) records estimated using dynamic heating methods.

#### 3.3.3 original_duration & duration_category

We recorded 19 different heating durations reported across all studies before categorizing them into four groups. We placed 39% (*n* = 1246) of records into the (0–5] minute duration category, 23% (*n* = 740) of records in the (5–25] minute category, 36% (*n* = 1152) of records in the (25–60] minute category and < 1% (*n* = 27) of records in the (60‐inf] minute category. Of the studies we included in our database, heat treatment durations ranged from a few seconds in dynamic heating rates (e.g., O'Sullivan et al. 2017) and up to 12 h (Mooney and Billings 1961), but durations > 60 min were relatively rare.

#### 3.3.4 original_recovery and recovery_category

We recorded 25 different original recovery times reported among studies and categorized them into six groups. The [0–1] minute recovery time category represented 43% (*n* = 1277) of these records, the (1–15] minute recovery time category represented < 1% (*n* = 11), the (15–720] minute recovery time category represented 3% (*n* = 126), the (720–1440] minute recovery time category represented 39% (*n* = 1273), the (1440–2880] minute recovery time category represented 3% (*n* = 8), and the (2880‐inf] minute recovery time category represented 10% (*n* = 312).

#### 3.3.5 Method

We reported five different physiological methods used to estimate heat tolerance. These methods include cell death, electrolyte leakage, FvFm, fo‐rise, and protoplasmic streaming. Heat tolerances assessed with cell death represent 15% (*n* = 475) of the database, fo‐rise method represents 37% (*n* = 1195), FvFm represents 42% (*n* = 1339), electrolyte leakage represents 5% (*n* = 147), and protoplasmic streaming represents 1% (*n* = 41) of records. The highest heat tolerance was assessed using cell death, and the lowest was assessed using the fo‐rise method.

#### 3.3.6 original_term & HT_standardized

We compiled 59 distinct terms used to describe heat tolerance estimates and categorized them into three main types: Tmax, T50, and Tcrit (Table S1). We assigned 30 distinct terms to the Tcrit category, 20 different terms as T50, and 9 different terms classified as Tmax.

### 3.4 Biogeographical Data

**FIGURE 3:**
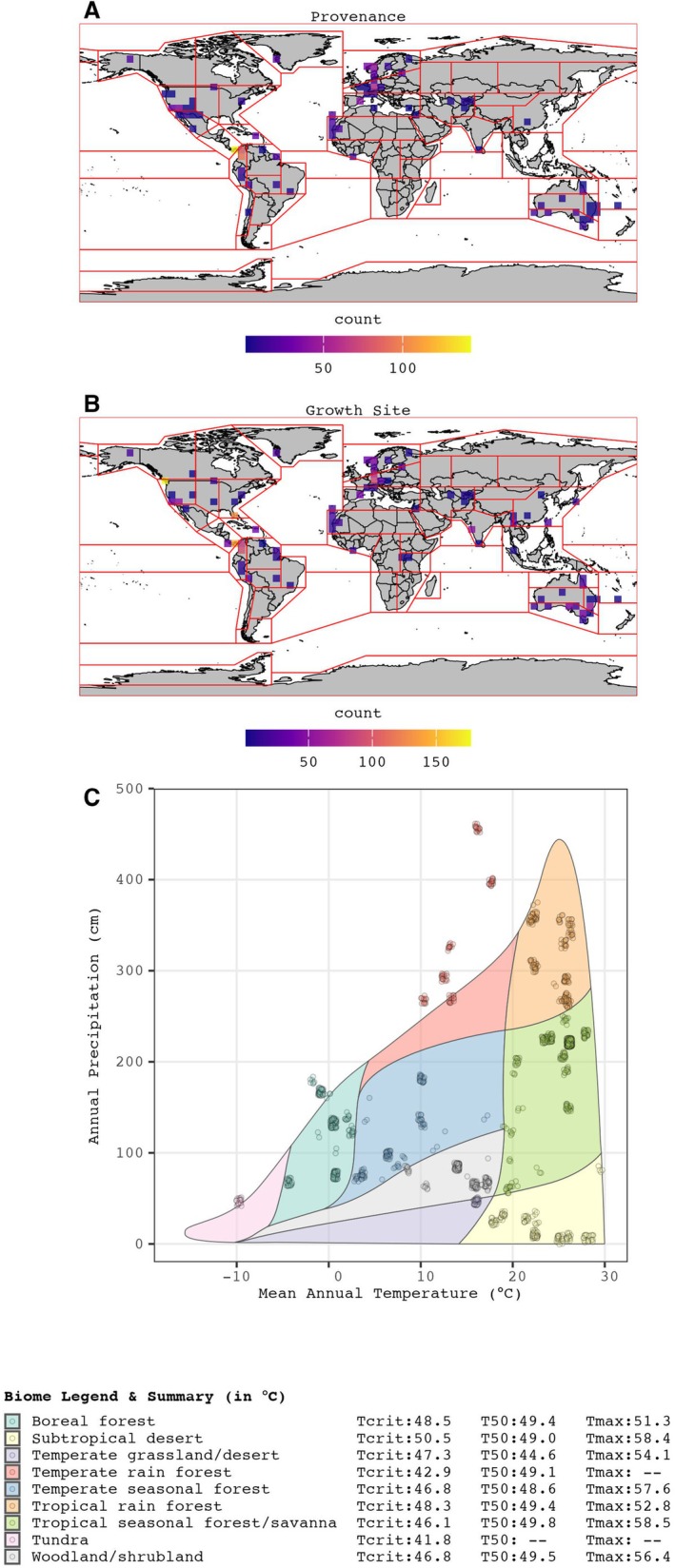
(A) Species sample size within each 500 × 500 km pixel with provenance data for each IPCC region. (B) Species sample size within each 500 × 500 km pixel with growth site data for each IPCC region. Pixel color indicates the number of records per IPCC region for A and B. (C) Biome representation of records in our database. Points represent heat tolerances records mapped to growth site climates after filtering for individuals grown in situ or common gardens with known provenance data (points jittered to illustrate sample sizes).

We recorded 1519 records with provenance coordinates (Figure 3A), 2913 records with growth site coordinates (Figure 3B), and 1322 heat tolerance records with provenance and growth coordinates that represented data from individuals grown in situ or in common garden environments. These 1322 records corresponded to 872 species from 46 sources and were used to calculate mean heat tolerances for IPCC regions and Whittaker biomes.

Estimates of Tcrit were available for 24 of the 58 IPCC regions, T50 was available for 21 regions, and Tmax was available for 11 regions (Table S2). Central‐American IPCC regions were the best represented in our dataset (*n* = 305) followed by South American (*n* = 279) and European (*N* = 61) regions. Over 50% of IPCC regions are not represented by any heat tolerance estimates in our database even when only provenance or growth location data are considered. Estimates of Tcrit were available for all 9 Whittaker Biomes, T50 were available for all biomes except the tundra, and at least one estimate of Tmax was available for all biomes except the tundra and temperate rainforests (Figure 3C). We reported the averages of each heat tolerance metric regardless of method in Table S5 for IPCC regions and in Figure 3C for biomes.

### 3.5 Cultivation Data

We collected agricultural status and growth condition metadata associated with each heat tolerance record. We classified 108 species (6% of all species in our dataset) as having agricultural or cultivated status, which accounted for 215 heat tolerance records (0.6% of all records). The four most frequently represented agricultural species were *Pisum sativum* (*n* = 9), *Mangifera indica* (*n* = 6), *Prunus avium* (*n* = 6), and *Zea mays* (*n* = 6). The three most common agricultural plant families were Fabaceae (*n* = 32), Poaceae (*n* = 26), and Solanaceae (*n* = 16). Our database contains 1721 in situ, 976 common garden, 248 greenhouse, and 73 growth chamber growth heat tolerance records. We were unable to obtain growth conditions for 179 records.

### 3.6 Bibliometric Data

We collected data from 131 different studies published from 1935 to 2024. Four studies contributed nearly one‐third of all records (33.1%) of the database records: Bison and Michaletz (2024, *n* = 354); Slot et al. (2021, *n* = 288); Perez and Feeley (2020, *n* = 246), and O'Sullivan et al. (2017, *n* = 168) However, 38.0% of all records in the database are from studies (*n* = 116) that report fewer than 50 records.

### 3.7 Correlations Among Methods

Only 58 pairwise comparisons were possible of the 2415 unique combinations (excluding self‐correlations) across all full methods in our database. We observed 15 statistically significant (*p* ≤ 0.05) correlations among 15 different methods for measuring heat tolerance (Table 3). The majority of the correlations we observed were between metrics of fluorescence, and methods that differed in one procedural step or standardized term (i.e., Tcrit, T50, or Tmax), but were otherwise identical. Notable significant correlations were observed between fo‐rise and cell death methods (Table 3: methods 7 & 8 and 8 & 9). All 2415 pairwise correlations are provided in Table S6.

**TABLE 3:** Significant correlations between heat tolerance methods with *p* ≤ 0.05 (BH adjusted *p*‐values).

Method 1 ID	Method 2 ID	Method 1	Method 2	Correlation summary		
				*r*	*p*	*n*
1	22	Static (5–25] (720–1440] FvFm T50	Static (5–25] (1440–2880] FvFm T50	0.934	0.00e+00	43
14	15	Static (25–60] (1440–2880] FvFm T50	Static (25–60] (1440–2880] FvFm Tcrit	0.908	0.00e+00	14
7	10	Dynamic (0–5] [0–1] fo‐rise Tmax	Dynamic (0–5] [0–1] fo‐rise T50	0.905	0.00e+00	66
9	10	Dynamic (0–5] [0–1] fo‐rise Tcrit	Dynamic (0–5] [0–1] fo‐rise T50	0.887	0.00e+00	70
24	58	static (5–25] (15–720] FvFm T50	static (5–25] (15–720] FvFm Tmax	0.810	0.00e+00	22
8	9	Static (25–60] NA cell death T50	Dynamic (0–5] [0–1] fo‐rise Tcrit	0.787	0.00e+00	26
7	32	Dynamic (0–5] [0–1] fo‐rise Tmax	Static (60‐inf] [0–1] FvFm T50	0.727	1.28e‐02	15
10	32	Dynamic (0–5] [0–1] fo‐rise T50	Static (60‐inf] [0–1] FvFm T50	0.720	1.32e‐02	15
38	40	Static (25–60] NA FvFm T50	Static (25–60] NA FvFm Tmax	0.707	1.55e‐02	15
9	32	Dynamic (0–5] [0–1] fo‐rise Tcrit	Static (60‐inf] [0–1] FvFm T50	0.706	1.28e‐02	16
9	14	Dynamic (0–5] [0–1] fo‐rise Tcrit	Static (25–60] (1440–2880] FvFm T50	0.671	3.33e‐02	14
7	8	Dynamic (0–5] [0–1] fo‐rise Tmax	Static (25–60] NA cell death T50	0.545	1.28e‐02	29
1	33	Static (5–25] (720–1440] FvFm T50	Static (5–25] (720–1440] FvFm Tmax	0.511	2.65e‐02	27
7	9	Dynamic (0–5] [0–1] fo‐rise Tmax	Dynamic (0–5] [0–1] fo‐rise Tcrit	0.224	8.29e‐04	288
1	29	Static (5–25] (720–1440] FvFm T50	Static (5–25] (720–1440] FvFm Tcrit	0.154	2.54e‐02	320

*Note:* Method IDs only correspond to this table.

## 4 Discussion

### 4.1 Data Acquisition

We compiled the largest database of heat tolerances for terrestrial plants to date. Our database doubles the number of species with heat tolerance data and more than triples the total number records compared to previous datasets for terrestrial plants. Although our database overlaps with previous datasets, it provides additional details describing measurement methods, biogeographical locations and cultivation information they excluded.

Our database can be distinguished from the other two existing databases by comparing the references in common. For a given reference shared between our database and the other two, our approach tended to procure more records per reference than other datasets. This is likely explained by our collection procedures that focused on collecting multiple different methods of measuring heat tolerances, while previous datasets did not, or collated heat tolerances with contrasting approaches. The other datasets do report more species and heat tolerance records when they include physiological data that we omit, like carbon assimilation or respiration. We excluded these types of physiological data because they are assessed with fundamentally different approaches but remain important higher‐order processes valuable for understanding the broader biological importance of the heat tolerances.

Our approach for locating references differed from the GlobTherm and Lancaster and Humphreys, which likely influenced the number of references and records we procured for our dataset. The GlobTherm and Lancaster and Humphreys databases used Google and a combination of Google and Web of Science search engines to locate references for their databases, respectively. The T. M. Perez (2019) dataset was created a year after the GlobTherm dataset and a year before the Lancaster and Humphreys dataset, but provides an important point of comparison. Like the datasets we present here, the T. M. Perez (2019) dataset relied on traditional forward and backward citation searches, but resulted in more references and more records than either the GlobTherm or Lancaster and Humphreys datasets combined. It is possible our approach is biased since we effectively sourced data from one network of articles linked together via citations. However, the greater number of records in our database compared to those from search engines refutes this idea. Given that search algorithms are routinely altered and produce different results at different times, our approach suggests search engine outputs can likely be augmented with traditional forward and backward citation searches to broaden the coverage of data collection opportunities.

### 4.2 Taxonomic Data

Although our database is the most comprehensive compilation of plant heat tolerance estimates to date, it represents only a small fraction of global plant diversity. The 1768 taxonomic entities included 1001 genera and 250 families, yet this coverage is extremely limited when compared with the estimated > 350,000 vascular plant species worldwide and their families and genera (Antonelli et al. 2023). The inclusion of a very few moss species highlights that nonvascular plants remain especially understudied. This taxonomic bias underscores both the need for caution when extrapolating our results to all plants and the opportunity for future work to expand heat tolerance measurements across underrepresented clades and functional types.

### 4.3 Methodological Data

The 67 different methods in our database equate to one novel methodological approach for measuring heat tolerances being reported from every other study we reviewed. Similarly, the 59 different terms we observed equate to more than one novel term reported for every two studies we reviewed. These results underscore the lack of standardized approaches in heat tolerance literature noted in other studies (Geange et al. 2021), which may contribute to hindering the ability to link heat tolerances with higher‐order ecological processes.

#### 4.3.1 HT

All of the heat tolerances that we report are provided in degrees Celsius (°C). Units of temperature in heat tolerances can be easily related to leaf and atmospheric temperatures relevant for understanding broader aspects of plant ecophysiology. However, there are instances of heat tolerances reported in other units such as the duration required for damage to be observed (Chen et al. 1982). The interacting effects of temperature treatment and duration on heat tolerance estimates provide a more nuanced and perhaps less biased assessment for relating plant physiology to higher‐order biological phenomena (Cook et al. 2024).

#### 4.3.2 static_dynamic

It is unclear how different methods of heating may influence physiology and resulting heat tolerances. The specific heating tools or methods used for static or dynamic heating are not recorded in this database, but commonly include hot water baths with or without direct contact with water (Colombo and Timmer 1992), a Peltier device (e.g., Godoy et al. 2011), and even steam‐saturated high air temperatures (e.g., Sapper 1935). Dynamic heat treatments continuously heat leaves and are more representative of how leaves experience increases in temperature in situ, but confound the effect of temperature treatment and duration on heat tolerances (Krause et al. 2010). Dynamic heating treatments that differ in the rate of heating have also been shown to influence heat tolerances in some species, further complicating heat tolerance treatments among studies (Arnold et al. 2021).

The effect of different heating rates on heat tolerances has not been fully investigated, but may be an important consideration for future research given leaf tissue heating rates may be influenced by a thermal time constant theorized to help modulate leaf temperature and avoid thermal damage (Leigh et al. 2012).

Static heating procedures do not confound the effects of temperature treatment and duration, but may not represent the cumulative effects of temperature on physiological processes. Ongoing research on the cumulative thermal stress provides a potential framework to harmonize heat tolerances estimated with static and dynamic heating approaches (Cook et al. 2024).

#### 4.3.3 original_duration & duration_category

The duration of heat treatments varies extensively in our data which may bias heat tolerance estimates. For example, the relationship between heat treatment duration and heat tolerances has been described using an exponential decay function (Gauslaa 1984; Colombo and Timmer 1992; Cook et al. 2024). This modeled relationship suggests that 30‐s and 5‐min heat treatments with identical static temperature treatments can result in at least a 4.5°C difference in heat tolerance (Colombo and Timmer 1992). Conversely, a 4.5°C difference in heat tolerance can also be observed between 20 and 160 min durations based on this same modeled relationship. This suggests that long‐duration heat tolerances may reduce the magnitude of measurement errors during heat tolerance assessments.

However, heat treatments longer than 20 min may allow acclimation of heat tolerances and introduce bias (Havaux 1993). Ultimately, different species may exhibit different shapes of their exponential decay curves in response to heat treatment duration and that collating heat tolerances with different durations may introduce bias (Gauslaa 1984; Cook et al. 2024). We advocate for caution when drawing conclusions from heat tolerance data aggregated from different heat treatment durations.

#### 4.3.4 original_recovery & recovery_category

Recovery times allow for repair following heat treatment so irreversible damage can be assessed. The recovery times in our database range from nonexistent—in the case of fo‐rise methods representing reversible damage—to minutes and weeks in studies when measuring cell death. Different recovery times may have dramatic effects on heat tolerance estimates. For example, assessing heat damage 15 min and 1440 min (24 h) after heat treatment resulted in a 2.3°C increase in the heat tolerances of *Ficus insipida* when assessed with FvFm chlorophyll fluorescence (Krause et al. 2010). Conversely, heat tolerance may decrease with recovery times when assessed using other methods like cell death (Mooney and Billings 1961; Krause et al. 2015; Winter et al. 2024). This highlights that different physiological processes may require different recovery periods, and that recovery time reflects an underlying physiological recovery process in addition and a procedural step.

Although we report original and standardized recovery times, the recovery of the corresponding physiological processes being measured is not implicit. Ultimately, even heat tolerances of different physiological processes that are assessed with identical methods may reflect different levels of reversible or irreversible damage. Heat tolerance data with differing recovery times may indeed indicate ranges of recovery, which we do not report.

#### 4.3.5 Method

The five physiological methods of assessing heat damage we recorded were cell death, electrolyte leakage, FvFm, fo‐rise, and protoplasmic streaming. These methods may largely reflect approaches used to report heat tolerances in units of temperature that we targeted. As such, our database should not be considered exhaustive of all approaches used to assess heat tolerances in photosynthetic tissues. For example, we intentionally excluded important physiological processes like carbon assimilation and respiration because they are treated elsewhere (e.g., Kumarathunge et al. 2019; Atkin et al. 2015). Future efforts collating disparate plant databases and physiological methods may advance heat tolerance research by revealing novel links in physiological responses.

#### 4.3.6 original_term & HT_standardized

We categorized 54 distinct terms for heat tolerance into Tmax, T50, or Tcrit. Our standardized terms for heat tolerance are heuristics that make the excessive terminology for heat tolerances more tractable for summary and descriptive statistics. Importantly, this artificially reduces the complexity for analyzing many different methods and may compromise the precision of the metrics that were originally reported.

Our database highlights the extensive and often inconsistent terminology used to describe plant heat tolerance, which partly reflects the diversity of measurement approaches. One notable exception is T50, which is consistently defined as the temperature causing a 50% change in a physiological response variable. We suspect the widespread use of T50 stems from its standardized definition based on percent change relative to baseline conditions, which makes it broadly applicable across different physiological metrics. In contrast, the terms Tcrit and Tmax are more ambiguous, often lacking a clear association with a specific threshold of physiological change or damage. Given that Tcrit and Tmax are categories of heterogeneous metrics, they may exhibit more variation when analyzed using our standardized format compared to the T50 category.

Despite extensive variation in methods and terminology, standardized approaches may not be warranted. It is possible that the lack of standardized methods reflects the lack of coordination with other physiological processes or plant performance, and standardized methods may stifle innovation or novel insights. Standardization may not be appropriate since different approaches probe distinct physiological processes. Instead, we advocate for uniform reporting terminologies of heat tolerances regardless of the methods used as a way to reduce the proliferation of terminology and promote comparisons among studies. For example, reporting Tcrit, T50, and Tmax heat tolerance metrics together and along with the percent damage or change associated with the Tcrit and Tmax heat tolerances is likely to facilitate interpretability of heat tolerance estimates across studies. This approach will help preserve the utility of legacy methods without discouraging novel approaches with clear physiological relevance.

### 4.4 Biogeographical Data

We used geolocation data to map heat‐tolerance records to IPCC regions and Whittaker biomes (Figure 1F; Table S5). These summaries are intended to illustrate the geographic and climatic coverage of the dataset, and to provide a coarse reference for broad‐scale ecological or modeling applications. Since these data combine diverse species, methods, and physiological measurements, they should be interpreted as heuristic rather than precise biome or IPCC‐level estimates. Importantly, the summaries make sampling gaps transparent and offer an initial benchmark for refinement and incorporating heat tolerance into broader‐scale ecosystem analyses. The averaging across methods masks important physiological differences, but these composites remain useful given the lack of method‐ or species‐specific estimates for most regions.

Despite the size of our database, geographic coverage of plant heat tolerances remains extremely limited. Fewer than half of the broadly defined IPCC regions are represented, and finer‐scale ecoregions would show even greater gaps. This limited scope means that generalizations about global patterns in heat tolerance may be hard to achieve. This issue is exacerbated given that heat tolerances may acclimate, which means estimates from location may not be representative of those for the same species in different micro‐ or macroclimates (Lancaster and Humphreys 2020; Zhu et al. 2018; Kullberg and Feeley 2024). Expanding sampling efforts, particularly those that link heat tolerance to broader measures of organismal performance would likely facilitate methodological standardization and should be a global priority.

### 4.5 Cultivation Data

We collected data from studies that reported heat tolerances in degrees Celsius, which allows for standardized comparisons across physiological processes, species, and abiotic factors such as microhabitat or climate. This selection criteria may have biased against agricultural species that often have heat tolerances measured in units of crop yield. Since crop yield tends to be species‐specific and in different units, they were incompatible with our database. Incorporating agricultural data into our database may become feasible if crop yield can be reliably linked to temperature‐based measures of heat tolerance.

Our data includes growth conditions, which enable users to determine whether plants were coupled or decoupled from ambient environments. However, we did not include other variables commonly associated with growth conditions such as night‐ versus daytime temperatures, light regimes, or watering treatments, all of which are known or hypothesized to influence heat tolerance estimates. Incorporating these variables in future efforts may help explain additional variation in the heat tolerance data.

### 4.6 Bibliometric Data

Our bibliometric analysis highlights the advantages of forward and backward citation searching for assembling heat tolerance records. Our approach allowed us to systematically identify relevant empirical studies, including older or less visible work that might not appear in keyword searches. This strategy yielded a greater number of references, species, and records than comparable datasets even when we drew on overlapping sources. Nevertheless, gray literature and non‐English publications may be underrepresented. However, we hope that this database will help surface additional sources and mitigate any biases in our collection approach.

The four studies that contribute roughly one third of all records in our database may introduce biogeographic and methodological biases into broader analyses of plant heat tolerances. Three of these studies represent large heat‐tolerance datasets derived from a single site (Bison and Michaletz 2024; Perez and Feeley 2020) or a small geographic region (Slot et al. 2021), leading to potential over‐representation of those areas. In addition, two of the studies (Bison and Michaletz 2024; Perez and Feeley 2020) were conducted in botanical gardens and include mixtures of native and non‐native species of unknown provenance. Such collections may alter observed trait vs. environment relationships compared to data collected in situ (but see Perez and Feeley 2020, Perez and Feeley 2021). Users of this database may need to account for the interacting effects of study location and methodological approach when conducting future analyses.

### 4.7 Correlations Among Methods

#### 4.7.1 Physiological Limitations

The majority of methods in our database showed no correlation. Heat tolerance estimates using fluorescence fo‐rise and FvFm methods did exhibit several correlations, which is expected for closely related physiological processes. We also observed correlations among cell death and fo‐rise methods, which may suggest coordinated underlying physiological mechanisms.

The correlations we found between fo‐rise and FvFm heat tolerances suggest that FvFm heat tolerances may also be coordinated with heat tolerances assessed using cell death. However, we found no correlation between FvFm and cell death heat tolerances, which is consistent with research intentionally investigating these two methods of assessing heat tolerance (Winter et al. 2024). This suggests that correlations between fo‐rise and cell death heat tolerances reveal related but distinct physiological processes from FvFm heat tolerances. Alternatively, the coordination we observed between fo‐rise and cell death heat tolerances in our study may have been erroneous.

The limited number of correlations we observed may simply result from the lack of standardized methods for assessing heat tolerances across studies. Inherent biases in the data we collected and did not account for in our database may also contribute to the lack of correlations we observed. For instance, we did not record metadata related to night or daytime growth temperatures, drought, light acclimation, or tissue and organismal ontogeny that may influence heat tolerance estimates. Alternatively, the lack of coordination we observed may suggest orthogonal physiological pathways.

The lack of coordination among different heat tolerances poses an important barrier for advancing heat tolerance research. Assuming that different metrics of heat tolerances are interchangeable may result in misleading conclusions until the mechanistic relationships among them are elucidated. Understanding how heat tolerances are coordinated is necessary to reveal the related physiological mechanisms, and how they may scale to different levels of biological complexity. Until such relationships are established, users of our database should exercise caution when conducting analyses with heat tolerances assessed with different metrics.

## 5 Conclusion

Plant heat tolerances are widely measured but hard to generalize due to inconsistent methods. We compiled the largest database to date, containing over 3000 records from over 1700 taxa, with standardized taxonomy, methodological, geographic, and cultivation metadata. We showed that heat tolerances measured with differing physiological methods may be related, but liklely capture information at different levels of biological organization and limits their ability to consistently scale to broader measures of plant performance. Our database highlights key gaps in geography and lineages to pursue in future investigation while providing a framework for disambiguating methods, evaluating their comparability, and linking heat tolerance to broader ecological and evolutionary processes.

To address these challenges, we encourage researchers to, at a minimum, report (1) the physiological response variable used to assess heat tolerance; (2) static or dynamic heating method; (3) the duration of heat treatment for static heat treatments or rate of heating for dynamic heat treatments; (4) the time allowed for recovery following heat treatment; and (5) the metric of heat tolerance calculated as a percentage of damage relative to any control treatment. We also encourage reporting additional sources of variation that are outlined in our methods section where applicable.

Although our database is primarily concerned with the effect of methodological variation on heat tolerance, myriad abiotic and biotic sources of variation have been reported. Ample evidence illustrates the effects of ambient temperatures (Havaux 1993; Daas et al. 2008; Zhu et al. 2018), light exposure (Valladares and Pearcy 1997, Krause et al. 2015, Slot et al. 2018), water regimes (Kullberg et al. 2026), and age (Yamada et al. 1996; Marias et al. 2017) on heat tolerance estimates. Quantifying the effect of these sources of variation on heat tolerance is likely to reveal new patterns in plant macroecology and climate vulnerability, yet no comprehensive quantitative review of such sources of variation in heat tolerance yet exists (but see Posch et al. 2025).

We are wary to recommend specific methodological approaches here for several reasons. For example, the approach used to assess heat tolerance is highly dependent on interests that are likely to vary among researchers. Furthermore, the limited evidence linking heat tolerances reported herein to broader metrics of plant performance precludes any objective justification for doing so. Instead, we recommend research that focuses on identifying the mechanistic relationships among heat tolerances of different physiological processes and the effect of these processes on higher‐order biological processes like growth, survival, or fecundity prior to any methodological coalescence.

## Author Contributions

**Timothy M. Perez:** conceptualization (equal), data curation (equal), formal analysis (equal), investigation (equal), methodology (equal), project administration (equal), resources (equal), validation (equal), visualization (equal), writing – original draft (equal), writing – review and editing (equal). **Alyssa T. Kullberg:** conceptualization (equal), data curation (equal), formal analysis (equal), investigation (equal), methodology (equal), resources (equal), writing – review and editing (equal). **Evan M. Rehm:** conceptualization (equal), data curation (equal), formal analysis (equal), investigation (equal), resources (equal), writing – review and editing (equal). **Kenneth J. Feeley:** conceptualization (equal), funding acquisition (equal), investigation (equal), methodology (equal), project administration (equal), resources (equal), writing – original draft (equal), writing – review and editing (equal).

## Funding

This work was supported by National Science Foundation DEB 2344948 to KJF and the Garden Club of America to TMP.

## Conflicts of Interest

The authors declare no conflicts of interest.

## Supporting information


**Table S1:** Data in Present study database that overlap with and the Globtherm database.
**Table S2:** Data in Present study database that overlap with and the Lancaster and Humphreys database.
**Table S4:** Summary statistics corresponding to the boxplot in main Figure 2.
**Table S6:** The 2415 pairwise correlations among heat tolerances in our database.

**Figure S1:** A diagram depicting how references from Perez (2019), Lancaster and Humphrey (2020), and the GlobTherm (Bennett 2018) databases were integrated into the present database.
**Table S3:** The categorized standardized terminology and the original terminology reported within our database. Superscripts correspond to references provided below.
**Table S5:** The mean heat tolerances for IPCC regions.

## Data Availability

All data are available from Dryad https://doi.org/10.5061/dryad.cjsxksnj2 with updatedversions located on https://tmoreperez.github.io/heat‐tolerance‐database/.
