# Supplementary material for: A Database of Plant Heat Tolerances and Methodological Matters
Source: Ecol Evol. 2026 Jun 17;16(6):e73813. doi: 10.1002/ece3.73813 (PMC13275996; doi:10.1002/ece3.73813)
Supplement: Supplementary file 2 — Figure S1: A diagram depicting how references from Perez (2019), Lancaster and Humphrey (2020), and the GlobTherm (Bennett 2018) databases were integrated into the present database. Table S3: The categorized standardized terminology and the original terminology reported within our database. Superscripts correspond to references provided below. Table S5: The mean heat tolerances for IPCC regions. [file ECE3-16-e73813-s002.docx]

Supporting Information:

**Section 1: Literature Search:**

The Perez 2019 dataset was cross-referenced against the Lancaster and Humphreys (2020) dataset, and incorporated into the current version of our dataset. Additional references were obtained through forward and backward references searches and their data was incorporated our dataset. We also cross-referenced the GlobTherm dataset and incorporated data from applicable references into our database. The resulting database we provide is termed Perez et al 2025.

Here, we describe how we incorporated data from references and how our collection procedures led to notable deviations in the data we report compared to other datasets. For example, the GlobTherm dataset contained 12 references corresponding to 73 heat tolerance records for 73 species after filtering for “Tmax” and “Streptophytes” (Table 1). Seven of these references were already common to the Perez 2019 dataset. These seven references corresponded to 222 heat tolerance records for 81, and 63 records from 62 species in the Perez 2019 and GlobTherm datasets, respectively (main text Table 2). We incorporated data from 4 new in the Globtherm dataset into our current dataset, which corresponded to 14 heat tolerances records for 11 species. One reference in the GlobTherm database was excluded because it provided data for algae and our study focuses on terrestrial plants.

Figure S1: A diagram depicting how references from Perez 2019, Lancaster and Humphrey (2020), and the GlobTherm (Bennett 2017) databases were integrated into the present database.

We filtered the Lancaster and Humphreys (2020) dataset to include data categorized as “Tmax”, which yielded 37 applicable references (a total of 689 species and 964 records; Table 1), of which 16 were common to the Perez 2019 dataset. The 16 overlapping references represented 716 records for 566 species and 778 records for 545 species for the Lancaster and Humphreys (2020) and Perez 2019, datasets respectively. We then incorporated data from 14 references unique to the Lancaster and Humphreys (2020) into our database, which corresponded to 128 new heat tolerance records for 77 species in our database (Table 2). We disqualified 7 references included in the Lancaster and Humphreys dataset because they had 1) data already incorporated into our database but sourced from other references, 2) did not have analogous physiological measurements (e.g. carbon assimilation or respiration data), or 3) could not be accessed for assessment.

***
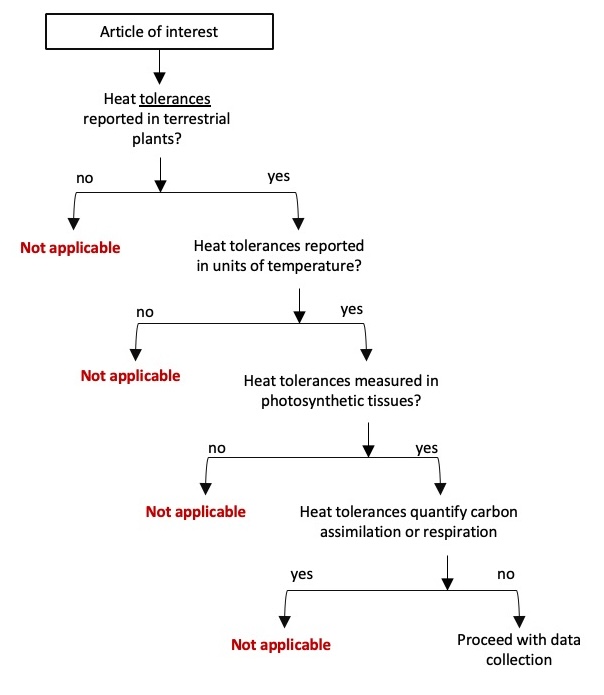
***

*Figure S2:* Diagram illustrating the criteria we used to collect data from articles

We followed a decision tree, illustrated in Figure S2, to determine if data were eligible to be included in database. This process generally involved reading the articles to clearly understand the data different studies were reporting, and in doing so provided references to other studies that we collected data from. Studies that we deemed eligible were reviewed more thoroughly for full data collection efforts that are described in the Methods sections of our main text.

***Section 2: Methodological data descriptions, results and discussion***

*Table S3:* The categorized standardized terminology and the original terminology reported within our database. Superscripts correspond to references provided below.

| **Categorized Heat Tolerance Terms** | **Original Heat Tolerance Terms** | **Number of Original Terms** |
| --- | --- | --- |
| Tmax | Ft100^97^, Lethal Temperature^130^, LT100^76,97^, t5^114^, T95^66,67,68,89,123,125,126^, TL100^73^, Tmax^10,57,128^, Tp^9,11,18^, TPH^18^ | 9 |
| T50 | 50% inhibition of stain uptake^29,93,94,115^, FT50 High^24^, FT50^97^, Fv/Fm T50^58^, H50^52^, heat tolerance^61^, hitzresistenz^53,71,72,82,83^, indirect damage^22^, killing temperature^20,119^, LT50 High^24^, LT50^3,8,9,11,13,14,15,16,38,39,69,73,74,81,91,97,99,103^, Maximal Temperature^104^, membrane permeability^79^, NA^4,5,101^, PSII thermotolerance^44,90^, R^54^, Resistance value^130^, T50^1,19,23,25,26,27,30,32,35,36,41,42,43,50,57,62,63,64,65,66,67,68,78,86,89,92,98,100,105,106,111,112,118,120,121,123,125,126,128^, Tl^64^, Warmeresistenz^6^ | 20 |
| Tcrit | Critical temperture^88^, F0T0^77,122^, F0Tc^131^, Fi^97^, Fluorescence rise temperature^108,109^, Fo increase^113^, H0^52^, heat resistance limit^46,47,48,49^, hitzeresistenz^7^, lethal^60^, LT10^69^, LT15^116^, LT5^16,17^, Lti^97^, Resistance limit^130^, streaming^2^, T-F0^12^, T15^19,37,41,50,92^, t2^31,114^, T5^66,67,68,89,123^, Tc break^75^, Tc^8,9,11,16,17,18,21,28,37,40,45,55,59,64,80,87,96,102,124,127,128^, TCH+TPH/2^18^, TCH^18^, Tcrit^10,34,56,66,84,85,86,95,100,111,112,117,121,125,126,129^, Tfr^51^, thermal damge^110^, Threshold temperature^33,70,107^, TL0^73,76^, TS20^57,58,128^ | 30 |

| **Reference list:** |
| --- |
| 1: Ahrens CW, Challis A, Byrne M, Leigh A, Nicotra AB, Tissue D, Rymer P. 2021. Repeated extreme heatwaves result in higher leaf thermal tolerances and greater safety margins. New Phytologist 232: 1212-1225 |
| 2: Alexandrov VY. 1964. Cytophysiological and Cytoecological Investigations of Heat Resistance of Plant Cells Toward the Action of High and Low Temperature. The Quarterly Review of Biology 39: 35-77 |
| 3: Balagurova, N., Drozdov, S. & Grabovik, S. 1996. Cold and heat resistance of five species of Sphagnum. Annales Botanici Fennici. 33, 33-37. |
| 4: Berry, J, Bjorkman, O. 1980. Photosynthetic Response and Adaptation to Temperature in Higher Plants. Annu. Rev. Plant Physiol. 31: 491-543 |
| 5: Biebl R, Maier. 1969. Tageslange und Temperaturresistenz. Osterreichische Botanische Zeitschrift, 117(2), 176-194 |
| 6: Biebl, R. 1964. Temperaturresistenz tropischer Pflanzen auf Puerto Rico. Protoplasma 59: 133-156 |
| 7: Biebl, R. 1967. √úber W√§rmehaushalt und Temperaturresistenz arktischer Pflanzen in Westgr√∂nland. Flora oder Allg. Bot. Zeitung. Abt. B, Morphol. und Geobot. 157: 327‚Äì354. Urban & Fischer. |
| 8: Bigras FJ. 2000. Selection of white spruce families in the context of climate change: Heat tolerance. Tree Physiology 20: 1227-1234 |
| 9: Bilger H-W, Schreiber U, Lange OL. 1984. Determination of leaf heat resistance: comparative and tissue necrosis methods. Oecologia 63: 256-262 |
| 10: Bison, N, Michaletz, S. 2024. Variation in leaf carbon economics, energy balance, and heat tolerance traits highlights differing timescales of adaptation and acclimation. New Phytologist https://doi.org/10.1111/nph.19702 |
| 11: Braun, V, Buchner, O, Neuner, G. 2002. Thermotolerance of Photosystem 2 of Three Alpine Plant Species Under Field Conditions. Photosynthetica 40: 587-595. Kluwer Academic Publishers. |
| 12: Bryant C, Harris RJ, Brothers N, Bone C, Walsh N, Nicotra AB, Ball MC. 2024. Cross-tolerance: Salinity gradients and dehydration increase photosynthetic heat tolerance in mangrove leaves. Functional Ecology 00: 1-13 |
| 13: Buchner O, Karadar M, Bauer I, Neuner G. 2013. A novel system for in situ determination of heat tolerance of plants: first results on alpine dwarf shrubs. Plant methods 9: 7 |
| 14: Buchner O, Neuner G. 2001. Determination of heat tolerance: A new equipment for field measurements. Journal of Applied Botany and Food Quality 75: 130-137 |
| 15: Buchner O, Neuner G. 2003. Variability of Heat Tolerance in Alpine Plant Species Measured at Different Altitudes. Arctic, Antarctic, and Alpine Research 35: 411-420 |
| 16: Buchner, O, Roach, T, Gertzen, J, Schenk, S, Karadar, M, St√∂ggl, W, Miller, R, Bertel, C, Neuner, G, Kranner, I. 2017. Drought affects the heat-hardening capacity of alpine plants as indicated by changes in xanthophyll cycle pigments, singlet oxygen scavenging, a-tocopherol and plant hormones. Environmental and Experimental Botany. 133: 159-177 |
| 17: Buchner, O, Stoll, M, Karadar, M, Kranner, I, Neuner, G. 2015. Application of heat stress in situ demonstrates a protective role of irradiation on photosynthetic performance in alpine plants. Plant. Cell Environ. 38: 812-26 |
| 18: Buchner, O. & Neuner, G. 2010. Freezing cytorrhysis and critical temperature thresholds for photosystem II in the peat moss Sphagnum capillifolium. Protoplasma 243, 63-71. |
| 19: Chaves, CJN, Leal, BSS, Lemos-Filho, JP de. 2018. How are endemic and widely distributed bromeliads responding to warming temperatures? A case study in a Brazilian hotspot. Flora 238: 110-118 |
| 20: Chen H-H, Shen Z-Y, Li PH. 1982. Adaptability of Crop Plants to High Temperatures Stress. Crop Science 22: 719 |
| 21: Choinski Jr, JS, Gould, KS. 2010. Immature leaves of Weinmannia racemosa are more heat tolerant than mature leaves based on differences in chlorophyll a fluorescence and solute leakage. New Zealand Journal of Botany. 48(3-4): 163-177 |
| 22: Colombo SJ, Timmer VR. 1992. Limits of tolerance to high temperatures causing direct and indirect damage to white spruce. Tree Physiology 11: 95-104 |
| 23: Cook, AM, Berry, N, Milner, KV, Leigh, A. 2021. Water availability influences thermal safety margins for leaves. Functional Ecology. 35: 2179-2189 |
| 24: Cunningham SC, Read J. 2006. Foliar temperature tolerance of temperate and tropical evergreen rain forest trees of Australia. Tree Physiology 26: 1435-1443 |
| 25: Curtis EM, Gollan J, Murray BR, Leigh A. 2016. Native microhabitats better predict tolerance to warming than latitudinal macro-climatic variables in arid-zone plants. Journal of Biogeography 43: 1156-1165 |
| 26: Curtis EM, Knight CA, Leigh A. 2019. Intracanopy adjustment of leaf-level thermal tolerance is associated with microclimatic variation across the canopy of a desert tree (Acacia papyrocarpa). Oecologia 189: 37-46 |
| 27: Curtis EM, Knight CA, Petrou K, Leigh A. 2014. A comparative analysis of photosynthetic recovery from thermal stress: A desert plant case study. Oecologia 175: 1051-1061 |
| 28: Daas C, Montpied P, Hanchi B, Dreyer E. 2008. Responses of photosynthesis to high temperatures in oak saplings assessed by chlorophyll-a fluorescence: inter-specific diversity and temperature-induced plasticity. Annals of Forest Science 65: 305 |
| 29: Didden-zopfy, B, & Nobel, P S. 1982. Opuntia bigelovii. Oecologia, 176-180 |
| 30: Docherty EM, Gloor E, Sponchiado D, Gilpin M, Pinto CAD, Junior HM, Coughlin I, Ferreira L, Junior JAS, da Costa ACL, Meir P, Galbraith D. 2022. Long-term drought effects on the thermal sensitivity of Amazon forest trees. Plant, Cell & Environment 46(1):185-198 |
| 31: Downton WJS, Berry JA, Seemann JR. 1984. Tolerance of Photosynthesis to High Temperature in Desert Plants. Plant Physiology 74: 786-790 |
| 32: Drake JE, Tjoelker MG, V?rhammar A, Medlyn BE, Reich PB, Leigh A, Pfautsch S, Blackman CJ, Lopez R, Aspinwall MJ, et al. 2018. Trees tolerate an extreme heatwave via sustained transpirational cooling and increased leaf thermal tolerance. Global Change Biology 24: 2390-2402 |
| 33: Epron D. 1997. Effects of drought on photosynthesis and on the thermotolerance of photosystem II in seedlings of cedar (Cedrus atlantica and C. libani ). Journal of Experimental Botany 48: 1835-1841 |
| 34: Esperon-Rodriguez, M, Power, SA, Tjoelker, MG, Marchin, RM, Rymer, PD. 2021. Contrasting heat tolerance of urban trees to extreme temperatures during heatwaves. Urban Forestry and Urban Greeing. 66:127387 |
| 35: Fadrique, B, Baraloto, C, Bravo-Avila, C, Feeley, K. 2022. Bamboo climatic tolerances are decoupled from leaf functional traits across an Andean elevation gradient. Oikos. e09229 |
| 36: Feeley, K, Martinez-villa, J, Perez, T, & Duque, AS. 2020. The thermal tolerances,distributions ,and performances of tropical montane tree species. Frontiers in Forests and Global Change 3: 1-11. https://doi.org/10.3389/ffgc.2020.00025 |
| 37: Froux F, Ducrey M, Epron D, Dreyer E, Froux F, Ducrey M, Epron D, Dreyer E, Rouxa FF, Ucreyb MD, et al. 2004. Seasonal variations and acclimation potential of the thermostability of photochemistry in four Mediterranean conifers. Ann. For. Sci.: 235-241 |
| 38: Garrett, TY, Huynh, C-V, North, GB. 2010. Root contraction helps protect the ‚Äôliving rock‚Äú cactus Ariocarpus fissuratus from lethal high temperatures when growing in rocky soil. Am. J. Bot. 97: 1951-1960 |
| 39: Gauslaa Y. 1984. Heat Resistance and Energy Budget in Different Scandinavian Plants. Holarctic Ecology 7: 5-78 |
| 40: Ghouil H, Montpied P, Epron D, Ksontini M, Hanchi B, Dreyer E. 2003. Thermal optima of photosynthetic functions and thermostability of photochemistry in cork oak seedlings. Tree Physiology: 1031-1039 |
| 41: Gimeno TE, Pias B, Lemos-Filho J, Valladares F. 2008. Plasticity and stress tolerance override local adaptation in the responses of Mediterranean holm oak seedlings to drought and cold. Tree Physiology: 87-98 |
| 42: Godoy O, Lemos-filho JP De, Valladares F. 2011. Invasive species can handle higher leaf temperature under water stress than Mediterranean natives. Environmental and Experimental Botany 71: 207-214 |
| 43: Hanley, PA, Arndt, SK, Livesley, SJ, Szota, C. 2021. Relating the climate envelopes of urban tree species to their drought and thermal tolerance. Science of the total environment. 753: 142012 |
| 44: Havaux M, Tardy F. 1996. Temperature-dependent adjustment of the thermal stability of photosystem II in vivo: possible involvement of xanthophyll-cycle pigments. Planta 198: 324-333 |
| 45: Havaux M. 1993. Rapid photosynthetic adaptation to heat stress triggered in potato leaves by moderately elevated temperatures. Plant, Cell & Environment 16: 461-467 |
| 46: Hellmuth EO. 1968. Eco-Physiological Studies on Plants in Arid and Semi-Arid Regions in Western Australia: I. Autecology of Rhagodia Baccata (Labill.) Moq. The Journal of Ecology 56: 319-344 |
| 47: Hellmuth EO. 1971 a. Eco-Physiological Studies on Plants in Arid and Semi-Arid Regions in Western Australia: V. Heat Resistance Limits of Photosynthetic Organs of Different Seasons, Their Relation to Water Deficits and Cell Sap Properties and the Regeneration Ability. The Journal of Ecology 59: 365-374 |
| 48: Hellmuth EO. 1971 b. Eco-Physiological Studies on Plants in Arid and Semi-Arid Regions in Western Australia: IV. Comparison of the Field Physiology of the Host, Acacia Grasbyi and its Hemiparasite , Amyema Nestor Under Optimal and Stress Conditions. Journal of Ecology 59: 351-363 |
| 49: Hellmuth EO. 1971 c. Eco-Physiological Studies on Plants in Arid and Semi-Arid Regions in Western Australia: III. Comparative Studies on Photosynthesis, Respiration and Water Relations of Ten Arid Zone and Two Semi-Arid Zone Plants Under Winter and Late Summer Climatic Condit. Journal of Ecology 59: 225-259 |
| 50: Hernandez, G, Perez, T, Vargas, O, Kress, W, Molina-Bravo, R, Cordero, R, Seemann, J, Garcia-Robledo, C. 2022 Evolutionary history constrains heat tolerance of native and exotic tropical Zingiberales. Functional Ecology 36:3073‚Äì3084 |
| 51: Huve K, Bichele I, Tobias M, Niinemets. 2006. Heat sensitivity of photosynthetic electron transport varies during the day due to changes in sugars and osmotic potential. Plant, Cell and Environment 29: 212-228 |
| 52: Ishikawa, M, Gusta, LV. 1996. Freezing and heat tolerance of Opuntia cacti native to the Canadian prairie provinces. Canadian Journal of Botany. 74:1890-1895 |
| 53: Kappen L. 1964. Untersuchungen fiber den Jahreslauf. der Frost-, Hitze- und Austrocknungsresistenz von Sporophyten einheimischer Polypodiaceen (Filicinae) Flora, 155, 123-166 |
| 54: Karschon R, Pinchas L. 1971. Variations In Heat Resistance of Ecotypes. Aust. J. Bot.: 261-272 |
| 55: Kitao M, Lei TT, Koike T, Matsumoto Y, Ang L-H, Tobita H, Maruyama Y. 2000. Temperature response and photoinhibition investigated by chlorophyll fluorescence measurements for four distinct species of dipterocarp trees. Physiologia Plantarum 109: 284-290 |
| 56: Kitudom, N, Fauset, S, Zhou, Y, Fan, Z, Li, M, He, M, Zhang, S, Xu, K, Lin, H. 2022. Thermal safety margins of plant leaves across biomes under a heatwave. Science of the Total Environment 806:150416 |
| 57: Knight CA, Ackerly DD. 2002. An ecological and evolutionary analysis of photosynthetic thermotolerance using the temperature-dependent increase in fluorescence. Oecologia 130: 505-514 |
| 58: Knight CA, Ackerly DD. 2003. Evolution and plasticity of photosynthetic thermal tolerance, specific leaf area and leaf size: Congeneric species from desert and coastal environments. New Phytologist 160: 337-347 |
| 59: Koniger, M, Harris, GC, Pearcy, RW. 1998. Interaction between photon flux density and elevated temperatures on photoinhibition in Alocasia macrorrhiza. Planta. 205:214-222 |
| 60: Konis, E. 1949. The Resistance of Maquis Plants to Supramaximal Temperatures. Ecology, 30: 425-429. https://doi.org/10.2307/1932445 |
| 61: Krause GH, Cheesman AW, Winter K, Krause B, Virgo A. 2013. Thermal tolerance, net CO2 exchange and growth of a tropical tree species, Ficus insipida, cultivated at elevated daytime and nighttime temperatures. Journal of Plant Physiology 170: 822-827 |
| 62: Krause GH, Winter K, Krause B, Virgo A. 2015. Light-stimulated heat tolerance in leaves of two neotropical tree species , Ficus insipida and Calophyllum longifolium. Functional Plant Biology 42: 42-51 |
| 63: Krause GH, Winter K, Krause B, Virgo A. 2016. Protection by light against heat stress in leaves of tropical crassulacean acid metabolism plants containing high acid levels. Functional Plant Biology 43: 1061-1069 |
| 64: Krause, G.H., Winter, K., Krause, B., Jahns, P., Garc‚Äôa, M., Aranda, J., et al. 2010. High-temperature tolerance of a tropical tree, Ficus insipida: Methodological reassessment and climate change considerations. Funct. Plant Biol. 37: 890-900 |
| 65: Krause, GH, Cheesman, AW, Winter, K, Krause, B, Virgo, A. 2013. Thermal tolerance, net CO2 exchange and growth of a tropical tree species, Ficus insipida, cultivated at elevated daytime and nighttime temperatures. Journal of Plant Physiology. 170:822-827 |
| 66: Kullberg AT, Coombs L, Soria Ahuanari RD, Fortier RP, Feeley KJ. 2023. Leaf thermal safety margins decline at hotter temperatures in a natural warming experiment in the Amazon. New Phytologist. doi: 10.1111/nph.19413 |
| 67: Kullberg AT, Feeley KJ. 2022. Limited acclimation of leaf traits and leaf temperatures in a subtropical urban heat island. Tree Physiology 42(11):2266‚Äì2281 |
| 68: Kunert, N, Hajek, P, Hietz, P, Morris, H, Rosner, S, Tholen, D. 2022. Summer temperatures reach the thermal tolerance threshold of photosynthetic decline in temperate conifers. Plant Biology. 24:1254-1261 |
| 69: Ladinig, U, Pramsohler, M, Bauer, I, Zimmermann, S, Neuner, G, Wagner, J. 2015. Is sexual reproduction of high-mountain plants endangered by heat. Oecologia 177:1195-2010 |
| 70: Ladjal M, Epron D, Ducrey M. 2000. Effects of drought preconditioning on thermotolerance of photosystem II and susceptibility of photosynthesis to heat stress in cedar seedlings. Journal of Experimental Botany 20: 1235-1241 |
| 71: Lange OL, Lange R. 1963. Untersuchungen fiber Blattemperaturen, Transpiration und Hitzeresistenz an Pflanzen mediterraner Standorte (Costa brava, Spanien). Flora 153: 387-425 |
| 72: Lange OL. 1959. Untersuchungen tiber Warmehaushalt und Hitzeresistenz mauretanischer Wtistenund Savannenpflanzen. Flora oder Allgemeine botanische Zeitung, 147: 595-651 |
| 73: Larcher W, Holzner M, Pichler J. 1989. Temperaturresistenz inneralpiner Trockenrasen. Flora 183: 115-131 |
| 74: Larcher W, Kainmuller C, Wagner J. 2010. Survival types of high mountain plants under extreme temperatures. Flora: Morphology, Distribution, Functional Ecology of Plants 205: 3-18 |
| 75: Larcher W, Wagner J, Thammathaworn A. 1990. Effects of Superimposed Temperature Stress on in vivo Chlorophyll Fluorescence of Vigna unguiculata under Saline Stress. Journal of Plant Physiology 136: 92-102 |
| 76: Larcher W, Wagner J. 1976. Temperaturgrenzen der CO2-Aufnahme und Temperaturresistenz der Blaetter von Gebirgspflanzen im vegetationsaktiven Zustand. Oecologia Plantarum 11: 361-374 |
| 77: Larcher, W, Wagner, J, Lutz C. 1997. The effect of heat on photosynthesis, dark respiration and cellular ultrastructure of the arctic-alpine psychrophyte Ranunculus glacialis 34: 219-232 |
| 78: Leon-garcia, IV, & Lasso, E. 2019. High heat tolerance in plants from the Andean highlands_: Implications for paramos in a warmer world. PLOS One, 10(14), e0224218 |
| 79: Liu, Y., Cao, T. & Glime, J. M. 2003. The Changes of Membrane Permeability of Mosses under High Temperature Stress. The Bryologist 106, 53-60 . |
| 80: Logan BA, Monson RK. 1999. Thermotolerance of Leaf Discs from Four Isoprene-Emitting Species Is Not Enhanced by Exposure to Exogenous Isoprene. Plant Physiology 120: 821-826 |
| 81: Loik ME, Harte J. 1996. High-Temperature Tolerance of Artemesia tridentata and Potentilla gracilis. Oecologia 108: 224-231 |
| 82: Losch R. 1980. Die Hitzeresistenz der Pflanzen des kanarischen Lorbeerwaldes. Flora 170: 456-465 |
| 83: Maier R. 1971. Einflub von Photoperiode und Einstrahlungsst≈†rke auf die Temperaturresistenz einiger Samenpflanzen. ‚Ä¶sterreichische Botanische Zeitschrift, 119: 306-322 |
| 84: Marchin, RM, Backes, D, Ossola, A, Leishman, MR, Tjoelker, MG, Ellsworth, DS. 2021. Extreme heat increases stomatal conductance and drought-induced mortality risk in vulnerable plant species. Global Change Biology. 28: 1133-1146 |
| 85: Marchin, RM, Esperon-Rodriguez M, Tjoelker, MG, Ellsworth, DS. 2022. Crown dieback and mortality of urban trees linked to heatwaves during extreme drought. Science of the Total Environment 850: 157915 |
| 86: Marias, DE, Meinzer, FC, Woodru, DR, Mcculloh, KA. 2016. Thermotolerance and heat stress responses of Douglas- fi r and ponderosa pine seedling populations from contrasting climates. Tree Physiology, 301-315. https://doi.org/10.1093/treephys/tpw117 |
| 87: Methy, M, Gillon, D, Houssard, C. 1997. Temperature-induced changes of photosystem II activity in Quercus ilex and Pinus halepensis. Canadian Journal of Forest Research 27: 31-38 |
| 88: Meyer H, Santarius KA. 1998. Thermal Acclimation and Heat Tolerance of Gametophytes of Mosses. Oecologia 115: 1-8 |
| 89: Munchinger, IK, Hajek, P, Akdogan, B, Caicoya, AT, Kunert, N. 2023. Leaf thermal tolerance and sensitivity of temperate tree species are correlated with leaf physiological and functional drought resitance traits. Journal of Forestry Research 34: 63-76 |
| 90: Neuner G, Buchner O. 2013. Dynamics of Tissue Heat Toleranace and Thermotolerances of PS II in Alpine Plants (C. Lutz, Ed.). |
| 91: Neuner, G, Buchner, O, Braun, V. 2000. Short-Term Changes in Heat Tolerance in the Alpine Cushion Plant Silene acaulis ssp. excapa [All.] J. Braun at Different Altitudes. Plant Biology 2:677-683 |
| 92: Neves Chaves, CJ, Santos Leal, BS, Lemos-Filho, JP. 2015. Temperature modulation of thermal tolerance of a CAM-tank bromeliad and the relationships with acid accumulation in different leaf regions. Physiologia Plantarum 154:500-510 |
| 93: Nobel PS. 1988. Environmental biology of agaves and cacti. New York, New York: Cambridge University Press. |
| 94: Nobel, S, Geller, GN, Kee, SC, Departtnent, ADZ. 1986. Temperatures and thermal tolerances for cacti exposed to high temperatures near the soil surface. Plant Cell and Environment, 279-287 |
| 95: O'Sullivan OS, Heskel MA, Reich PB, Tjoelker MG, Weerasinghe KWLK, Penillard A, Zhu L, Egerton JJG, Bloomfield KJ, Creek D, et al. 2017. Thermal limits of leaf metabolism across biomes. Global Change Biology: 209-223 |
| 96: O'Sullivan OS, Weerasinghe KWLK, Evans JR, Egerton JJG, Tjoelker MG, Atkin OK. 2013. High-resolution temperature responses of leaf respiration in snow gum (Eucalyptus pauciflora) reveal high-temperature limits to respiratory function. Plant, Cell and Environment 36: 1268-1284 |
| 97: Offord CA. 2011. Pushed to the limit: Consequences of climate change for the Araucariaceae: A relictual rain forest family. Annals of Botany 108: 347-357 |
| 98: Okubo, N, Inoue, S, Ishii, HR. 2023. Tolerance and acclimation of the leaves of nine urban tree species to high temperatures. Forest. 14:1639 |
| 99: Ortiz, C, Cardemil, L. 2001. Heat-shock responses in two leguminous plants: a comparative study. J. Exp. Bot. 52: 1711-1719 |
| 100: Perez, TM, Feeley, KJ. 2020. Weak phylogenetic ad climatic constraints on photosynthetic heat tolerances. Journal of Biogeography. 48:91-100 |
| 101: Qiu, N, Lu, C. 2003. Enhanced tolerance of photosynthesis against high temperature damage in salt-adapted halophyte Atriplex centralasiatica plants. Plant, Cell and Environment 26:1137-1145 |
| 102: Ranney, TG, Ruter, JM. 1997. Foliar heat tolerance of three holly species (Ilex spp.): responses of chlorophyll fluorescence and leaf gas exchange to supraoptimal leaf temperatures. Journal of American Society of Horticultural Science. 122: 499-503 |
| 103: Reyes, M.A., Corcuera, LJ, Cardemil, L. 2003. Accumulation of HSP70 in Deschampsia antarctica Desv. leaves under thermal stress. Antarct. Sci. 15: 345-352. |
| 104: Sapper I. 1935. Versuche zur hitzeresistenz der pflanzen. Planta 23: 518-556. |
| 105: Sastry A, Barua D. 2017. Leaf thermotolerance in tropical trees from a seasonally dry climate varies along the slow-fast resource acquisition spectrum. Scientific Reports 7: 1-11. |
| 106: Sastry, A, Guha, A, Barua, D. 2017. Leaf thermotolerance in dry tropical forest tree species: relationships with leaf traits and effects of drought. AOB Plants 10:plx070 |
| 107: Schreiber U, Berry JA. 1977. Heat-induced changes of chlorophyll fluorescence in intact leaves correlated with damage of the photosynthetic apparatus. Planta 136: 233-238 |
| 108: Seemann JR, Berry JA, Downton WJS. 1984. Photosynthetic Response and Adaptation to High Temperature in Desert Plants A Comparison of Gas Exchange and Fluorescence Methods for Studies of Thermal Tolerance. Plant Physiol 75: 364-368 |
| 109: Seemann JR, Downton WJS, Berry JA. 1986. Temperature and Leaf Osmotic Potential as Factors in the Acclimation of Photosynthesis to High Temperature in Desert Plants. Plant Physiology 80: 926-930 |
| 110: Singsaas L, Lerdau M, Winter K, Sharkey TD. 1997. lsoprene lncreases Thermotolerance of Isopreme-Emitting Species. Plant physiology: 1413-1420 |
| 111: Slot M, Krause GH, Krause B, Hernandez GG, Winter K. 2019. Photosynthetic heat tolerance of shade and sun leaves of three tropical tree species. Photosynthesis Research 141:119-130 |
| 112: Slot, M, Cala, D, Aranda, J, Virgo, A, Michaletz, ST, Winter, K. 2021. Leaf heat tolerance of 147 tropical forest species varies with elevation and leaf functional traits, but not with phylogeny. Plant Cell Environ. 44: 2414-2427 |
| 113: Smillie RM, Gibbons GC. 1981. Heat tolerance and heat hardening in crop plants measured by chlorophyll fluorescence. Carlsberg Research Communications 46: 395-403 |
| 114: Smillie RM, Nott R. 1979. Heat Injury in leaves of alpine, temperate and tropical plants. Australian Journal of Plant Physiology 6: 135-141 |
| 115: Smith, SD, Didden-Zopfy, B, Nobel, PS. 1984. High-Temperature Responses of North American Cacti. Ecology, 65: 643-651. |
| 116: Snider, JL, Choinski, JS, Slaton, W. 2010. Juvenile leaves of Rhus glabra have higher photosynthetic thermal tolerance than mature leaves. Botany 88:286-289 |
| 117: Sonti, NF, Hallett, RA, Griffin, KL, Trammell, TLE, Sullivan, JH. 2021. Chlorophyll fluorescence parameters, leaf traits and foliar chemistry of white oak and red maple trees in urban forest patches. Tree Physiology 41: 269-679 |
| 118: Spiers, JA, Oatham, MR, Rostant, LV, Farrell, D. 2023. Determining the ecophysiological limits of a narrow niche tropical conifer tree (Podocarpus trinitensis) 43: 781-793 |
| 119: Srinivasam A, Hiroyuki T, Toshihiro S. 1996. Heat tolerances in food legumes as evaluated by cell membrane thermostability and chlorophyll fluorescence techniques. Euphytia 88: 35-45 |
| 120: Sumner, EE, Williamson, VG, Gleadow, RM, Wevill, T, Venn, SE. 2022. Acclimation to water stress improves heat tolerance to heat and freezing in a common alpine grass. Oecologia. 199:831-843 |
| 121: Tarvainen, L, Wittemann, M, Mujawamariya, M, Manishimwe, A, Zibera, E, Ntirugulirwa, B, Ract, C, Manzi, OJL, Andersson, MX, Spetea, C, Nsabimana, D, Wallin, G, Uddling, J. 2022. Handling the heat -- photosynthetic thermal stress in tropical trees. New Phytologist 233(1):236-250 |
| 122: Terzaghi WB, Fork DC, Berry JA, Field CB. 1989. Low and High Temperature Limits. Plant Physiology: 1494-1500 |
| 123: Tiwari, R, Gloor, E, Jonatar, W, Cruz, A, Marimon, BS, Marimon-junior, BH, √â Vitria, AP. 2020. Photosynthetic quantum efficiency in south-eastern Amazonian trees may be already affected by climate change. Plant Cell and Environment 1-12. https://doi.org/10.1111/pce.13770 |
| 124: Valladares F, Pearcy RW. 1997. Interactions between water stress, sun-shade acclimation, heat tolerance and photoinhibition in the sclerophyll Heteromeles arbutifolia. Plant, Cell And Environment 20: 25-36 |
| 125: Valliere, JM, Nelson, KC, MCastaneda Martinez, M. 2023. Functional traits and drought strategy predict leaf thermal tolerance. Conservation Physiology 11(1): coad085 |
| 126: Visakorpi, K, Manzanedo, RD, Gorlich, AS, Schiendorfer, K, AltermanttBieger, A, Gates, E, HilleRisLambers, J. 2023. Leaf-level resistance to frost, drought, and heat covaries across European temperate tree seedlings. Journal of Ecology 112: 559‚Äì574 |
| 127: Weng J, Lai M. 2005. Estimating heat tolerance among plant species by two chlorophyll fluorescence parameters. Photosynthetica 43: 439-444 |
| 128: Zhang JL, Poorter L, Hao GY, Cao KF. 2012. Photosynthetic thermotolerance of woody savanna species in China is correlated with leaf life span. Annals of Botany 110: 1027-1033 |
| 129: Zhu L, Bloomfield KJ, Hocart CH, Egerton JJG, O?Sullivan OS, Penillard A, Weerasinghe LK, Atkin OK. 2018. Plasticity of photosynthetic heat tolerance in plants adapted to thermally contrasting biomes. Plant Cell and Environment 41: 1251-1262 |
| 130: Zohar, Y, Waisel, Y, Karschon, R. 1981. Heat and cold resistance of Eucalyptus occidentalis Endl. Leaves and its relationship to soil water conditions. Australia Journal of Ecology 6:79-84 |
| 131: Zsofi Z, Varadi G, Balo B, Marschall M, Nagy Z, Dulai S. 2009. Heat acclimation of grapevine leaf photosynthesis: Mezo- and macroclimatic aspects. Functional Plant Biology 36: 310-322 |

**Section 3: IPCC Region heat tolerances**

Table S5: The mean heat tolerances for IPCC regions.

| Region | Tcrit | T50 | Tmax |  | Region | Tcrit | T50 | Tmax |
| --- | --- | --- | --- | --- | --- | --- | --- | --- |
| Arabian-Peninsula | NA | NA | NA |  | N.Pacific-Ocean | NA | NA | NA |
| Arabian-Sea | NA | NA | NA |  | N.South-America | 50.3 | NA | NA |
| Arctic-Ocean | NA | NA | NA |  | N.W.North-America | 41.8 | NA | NA |
| Bay-of-Bengal | NA | NA | NA |  | N.W.South-America | 48.3 | 49.2 | 56.2 |
| C.Australia | 53.8 | NA | NA |  | New-Zealand | 48.4 | NA | NA |
| C.North-America | 44.8 | NA | NA |  | Russian-Arctic | NA | NA | NA |
| Caribbean | NA | 47.9 | NA |  | Russian-Far-East | NA | NA | NA |
| Central-Africa | NA | NA | NA |  | S.Asia | 45.5 | 48.8 | NA |
| E.Antarctica | NA | NA | NA |  | S.Atlantic-Ocean | NA | NA | NA |
| E.Asia | NA | 44.7 | NA |  | S.Australia | 47.2 | NA | NA |
| E.Australia | 44 | 50.5 | 56.9 |  | S.Central-America | 46.9 | 50.1 | 52.8 |
| E.C.Asia | NA | NA | NA |  | S.E.Asia | NA | NA | NA |
| E.Europe | NA | NA | NA |  | S.E.South-America | NA | NA | NA |
| E.North-America | 47.4 | NA | NA |  | S.Eastern-Africa | NA | NA | NA |
| E.Siberia | NA | NA | NA |  | S.Indic-Ocean | NA | NA | NA |
| E.Southern-Africa | NA | NA | NA |  | S.Pacific-Ocean | 46.5 | 52.7 | 58.5 |
| Equatorial.Atlantic-Ocean | NA | NA | NA |  | S.South-America | NA | NA | NA |
| Equatorial.Indic-Ocean | NA | NA | NA |  | S.W.South-America | NA | NA | NA |
| Equatorial.Pacific-Ocean | NA | NA | NA |  | Sahara | NA | 49.2 | NA |
| Greenland/Iceland | 48.2 | NA | NA |  | South-American-Monsoon | 43.1 | 52.3 | 62.4 |
| Madagascar | NA | NA | NA |  | Southern-Ocean | NA | NA | NA |
| Mediterranean | NA | 50.5 | NA |  | Tibetan-Plateau | NA | NA | NA |
| N.Atlantic-Ocean | NA | NA | NA |  | W.Antarctica | NA | NA | NA |
| N.Australia | 46.2 | 52.2 | 58.3 |  | W.C.Asia | NA | NA | NA |
| N.Central-America | 53.4 | NA | NA |  | W.North-America | 47.7 | 47.6 | 56.8 |
| N.E.North-America | NA | NA | NA |  | W.Siberia | NA | NA | NA |
| N.E.South-America | 46.9 | 47.7 | NA |  | W.Southern-Africa | NA | NA | NA |
| N.Eastern-Africa | NA | NA | NA |  | West&Central-Europe | 49.4 | 51 | 53.1 |
| N.Europe | 47.2 | 47.8 | NA |  | Western-Africa | NA | 48.5 | NA |

We mapped occurrences to their growth site coordinates if they also had provenance data to estimate the mean heat tolerance of each IPCC region. This was done to highlight records with a full compliment of biogeographical data. We then calculated the mean heat tolerance regardless of method for all records within a given region. The mean Tcrit for IPCC regions ranged from 41.8˚C in NW North America to 53.8 in Central Australia, from 47.6˚C in W. North America to 52.7 in the Southern Pacific Ocean for T50, and from 52.8 in S. Central America to 62.4 in the South American Monsoon region for Tmax.
